# The Role of Short-Chain Fatty Acids, Particularly Butyrate, in Oncological Immunotherapy with Checkpoint Inhibitors: The Effectiveness of Complementary Treatment with *Clostridium butyricum* 588

**DOI:** 10.3390/microorganisms12061235

**Published:** 2024-06-19

**Authors:** Massimiliano Cazzaniga, Marco Cardinali, Francesco Di Pierro, Giordano Bruno Zonzini, Chiara Maria Palazzi, Aurora Gregoretti, Nicola Zerbinati, Luigina Guasti, Maria Rosaria Matera, Ilaria Cavecchia, Alexander Bertuccioli

**Affiliations:** 1 Scientific & Research Department, Velleja Research, 20125 Milan, Italy; maxcazzaniga66@gmail.com (M.C.); f.dipierro@vellejaresearch.com (F.D.P.); 2 Microbiota International Clinical Society, 10123 Torino, Italy; auroragregoretti@gmail.com (A.G.); jajamatera74@gmail.com (M.R.M.); ilaria.cavecchia@gmail.com (I.C.); alexander.bertuccioli@uniurb.it (A.B.); 3 Department of Internal Medicine, Infermi Hospital, AUSL Romagna, 47921 Rimini, Italy; marco.cardinali@uniurb.it; 4 Department of Biomolecular Sciences, University of Urbino Carlo Bo, 61122 Urbino, Italy; giordano.zonzini@uniurb.it; 5 Department of Medicine and Surgery, University of Insurbia, 21100 Varese, Italy; nicola.zerbinati@uninsubria.it (N.Z.); luigina.guasti@uninsubria.it (L.G.)

**Keywords:** immunotherapy, microbiota, immune checkpoints, butyrate

## Abstract

The discovery of immune checkpoints (CTLA-4, PD-1, and PD-L1) and their impact on the prognosis of oncological diseases have paved the way for the development of revolutionary oncological treatments. These treatments do not combat tumors with drugs “against” cancer cells but rather support and enhance the ability of the immune system to respond directly to tumor growth by attacking the cancer cells with lymphocytes. It has now been widely demonstrated that the presence of an adequate immune response, essentially represented by the number of TILs (tumor-infiltrating lymphocytes) present in the tumor mass decisively influences the response to treatments and the prognosis of the disease. Therefore, immunotherapy is based on and cannot be carried out without the ability to increase the presence of lymphocytic cells at the tumor site, thereby limiting and nullifying certain tumor evasion mechanisms, particularly those expressed by the activity (under positive physiological conditions) of checkpoints that restrain the response against transformed cells. Immunotherapy has been in the experimental phase for decades, and its excellent results have made it a cornerstone of treatments for many oncological pathologies, especially when combined with chemotherapy and radiotherapy. Despite these successes, a significant number of patients (approximately 50%) do not respond to treatment or develop resistance early on. The microbiota, its composition, and our ability to modulate it can have a positive impact on oncological treatments, reducing side effects and increasing sensitivity and effectiveness. Numerous studies published in high-ranking journals confirm that a certain microbial balance, particularly the presence of bacteria capable of producing short-chain fatty acids (SCFAs), especially butyrate, is essential not only for reducing the side effects of chemoradiotherapy treatments but also for a better response to immune treatments and, therefore, a better prognosis. This opens up the possibility that favorable modulation of the microbiota could become an essential complementary treatment to standard oncological therapies. This brief review aims to highlight the key aspects of using precision probiotics, such as *Clostridium butyricum*, that produce butyrate to improve the response to immune checkpoint treatments and, thus, the prognosis of oncological diseases.

## 1. Introduction

In recent decades, the effectiveness of oncological therapies has steadily improved, thereby improving the prognosis for many tumors. However, despite this, it remains evident that a significant proportion of patients continue to respond inadequately to oncological treatments [1]. Immunotherapy, one of the most innovative therapies in this regard, is no exception. Numerous efforts have been made to increase the effectiveness of immunotherapy and improve the prognosis of the disease. Among these efforts are various administration methods [2], which can have a significant impact on the effectiveness of immunotherapy itself. These methods influence the drug concentration, duration of action, immune response, and treatment tolerability. Immunotherapy has made significant progress in various forms of treatment, including cancer vaccines, adoptive cell transfer (ACT), and immune checkpoint inhibitors (ICIs). Considering the undeniable impact that the microbiota has demonstrated on the response to various oncological therapies, such as chemotherapy, immunotherapy, and radiotherapy [3], many studies are emphasizing how our ability to modulate the intestinal microbial composition could be beneficial. In particular, stimulating the production of certain beneficial compounds could help optimize therapeutic regimens in order to make them less toxic and more effective in terms of prognosis, especially concerning immunotherapy [4]. Authoritative publications state that the levels of short-chain fatty acids (SCFAs) present in our bodies, particularly butyrate, and/or our ability to stimulate their production, are strongly associated with the final treatment response [5].

## 2. The Fundamental Role of Immunity in Oncology

Our immune system, particularly the acquired immune response, better defined as specific or adaptive, is engaged when a pathogenic microorganism is present. This occurs following the targeted activation of B lymphocytes and T lymphocytes, which are specialized cells for this action. This mediated response can be triggered not only by infected cells (for example, by bacteria and viruses) but also by transformed cells—that is, “self” cells that will instead be recognized as “non-self” because, following the oncogenic transformation process, they begin to express “foreign” antigens on their surface [6]. Our immune system thus possesses the potential to control the indiscriminate growth of tumor cells, in addition to the ability to attack and destroy them through the action of T lymphocytes. The effectiveness of this response is highlighted by the presence of TILs (tumor-infiltrating lymphocytes) within the tumor mass, which is now widely accepted as a predictive biomarker for treatment responses and disease prognoses [7]. In practice, the presence of lymphocytes in the oncological mass, and especially the extent of this presence, represents a fundamental indicator for predicting the clinical course of the disease as well as the response to oncological treatments [8]. Much scientific evidence supports this notion. As early as 2003, Zhang et al. published a study in the *NEJM* that highlighted how the presence of TILs significantly influenced the treatment response and prognosis in advanced-stage ovarian cancer [9]. In recent decades, scientific evidence of this finding has been confirmed: for example, in breast cancer [10], as well as in lung and colon cancer, with some meta-analyses published in top-tier journals [11,12]. With these premises, it becomes quite clear and evident how a significant portion of the “battle against cancer” is played out at the onset of a neoplastic transformation, particularly by our body’s ability to respond to cellular oncologic insult, recognizing and eliminating potentially dangerous altered cells in a timely manner, regardless of the transforming factor. Especially with such a finely organized and efficient system, it remains somewhat incomprehensible how a large number of uncontrolled oncological developments are possible.

## 3. Immunotherapy in Oncology

**Figure 1:**
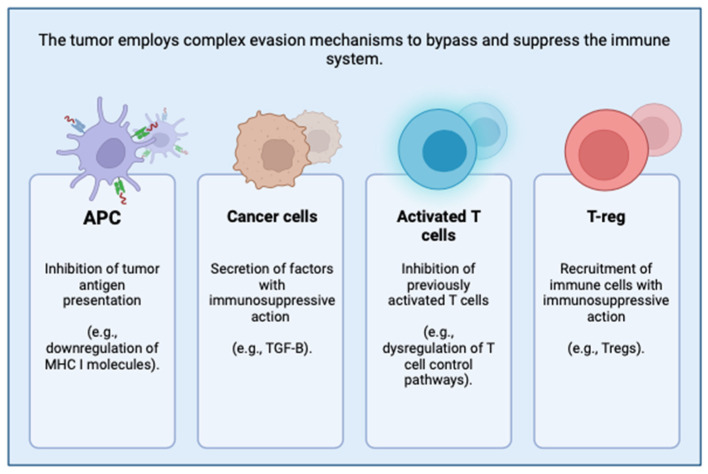
Mechanisms of tumor evasion from the immune system: inhibition of APCs, secretion of immunosuppressive factors (e.g., TGF-β), inhibition of previously activated cells, and recruitment of Tregs.

The answer to this question lies in the ability of our oncological cells to evade the immune response, but above all in an evolutionary limitation present in our immune system. Specifically, despite the optimal conditions in which tumor cells recognized by immune cells are destroyed and eliminated, all too often, carcinomatous cells are capable of activating multiple effective evasion phenomena that underlie treatment resistance and failure. Among the various mechanisms implemented by the tumor, the ability to inhibit the presentation of tumor antigens (APCs); the secretion of immunosuppressive factors (e.g., TGF-β), which inhibit the activation of cytotoxic T cells and NK cells, allowing the tumor to evade immune surveillance and progress easily; the inhibition of previously activated cells; and the recruitment of immunosuppressive and immunoregulatory immune cells (Tregs) are certainly important [13] (Figure 1).

However, the most important mechanism appears to be related to the action of immune checkpoints, exploiting a limitation of the system itself. In practice, evolution has created these “brakes” on our immune system to prevent it from proposing violent reactions (autoimmune) every time it is called into action. This evolutionary “subtlety”, which allows us to regulate the action of our defense system, protects us in most situations but proves counterproductive in cases where we would need to develop the full power of the immune system, such as in the presence of cancerous masses. In fact, it provides assistance to the tumor, which exploits this limitation to evade the response and proliferate uncontrollably. Therefore, oncological immunotherapy aims to unleash the power of the system by eliminating these brakes through the use of monoclonal antibodies that target these checkpoints (checkpoint inhibitors, or CPIs) [14].

Immunotherapy based on immune checkpoint inhibitors, such as anti-PD-1 and anti-PD-L1, has undoubtedly revolutionized the treatment of certain tumors, and it is becoming increasingly important in the innovation of oncological treatments. However, despite the promising results and undeniable clinical improvements achieved, it remains a fact that treatment with these drugs, alone or in combination, works in less than half of patients [15]. For this reason, researchers’ interest is increasingly focused on identifying factors that can predict the patient’s response to immunotherapy, in addition to the ability to increase the treatment’s effectiveness. Many studies have now highlighted the effectiveness of these treatments, particularly in certain forms of tumors, such as melanoma, lung carcinoma, and kidney carcinoma, showing a reduction in tumor mass but, above all, an increase in survival superior to that achieved with chemotherapy alone [16,17,18]. Despite these promising premises, a significant proportion of patients still do not respond to drugs, or they become resistant to them early on. Therefore, one of the most active “research sections” at present is focused on understanding the factors responsible for such issues and/or discovering elements that can help us bypass the problem. In this regard, much attention has been paid to the microbiota, and particularly to the quota of SCFAs that it can generate.

## 4. Short-Chain Fatty Acids (SCFAs)

**Figure 2:**
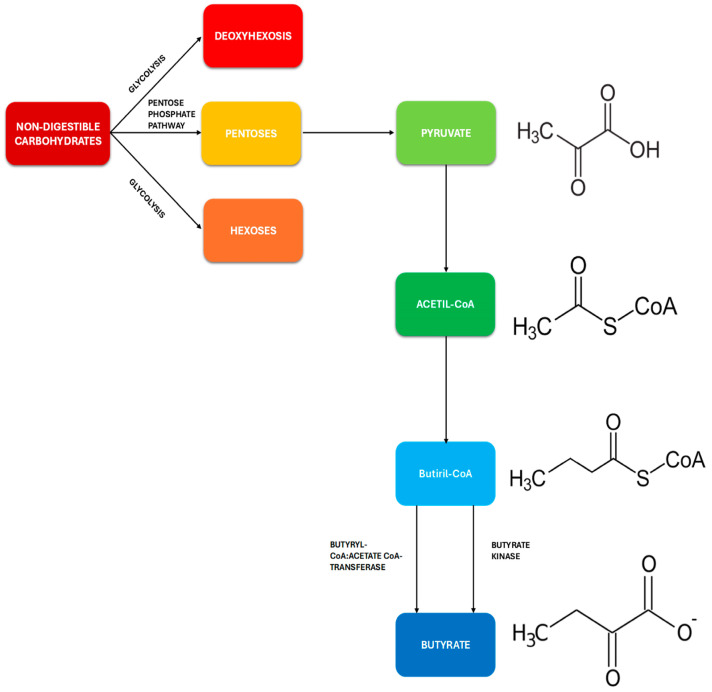
Synthesis of butyrate from carbohydrates with low or limited digestibility through fermentation mediated by intestinal bacteria [19].

Short-chain fatty acids (SCFAs) are defined as fatty acids with fewer than six carbon atoms. They are produced by the intestinal microbiota thanks to the ability of some bacterial groups to ferment certain undigested and absorbed nutrients in the small intestine, such as some carbohydrates with low or limited digestibility (polysaccharides, oligosaccharides, fibers, inulin, etc.). They are degraded into monosaccharide residues through the enzymatic action of certain bacterial groups, often associated with one another to increase fermentative capacity (a phenomenon known as cross-feeding), and then catabolized to form a compound called phospho-enol-pyruvate (PEP), a precursor of pyruvate and a determinant element from which SCFAs, and in particular the three most important ones (acetate, propionate, and butyrate), are derived through a series of complicated biochemical events [19] (Figure 2).

Among the bacterial groups suitable for this function, and, in particular, for generating butyrate (the short-chain fatty acid that we will extensively discuss in this work), the most important are certainly the Firmicutes phyla, especially those of the *Lachnospiraceae* and *Ruminococcaceae* genera, as they are capable of producing enzymes such as butyrate kinase and butyrate CoA transferase, which are responsible for the genesis of the majority of compounds [20].

These butyrate-producing bacterial genera contribute to maintaining the health of our microbiota, and among the strategies that can be adopted to promote intestinal wellbeing, undoubtedly, there is nutrition, which is capable of influencing our intestinal microbiota. In this regard, one of the most well-known dietary patterns is the Mediterranean diet, universally considered healthy due to the vast majority of its applications. Its distinctive features, including the use of olive oil, fish, and its abundance of fruits and vegetables, make it functional in controlling the glycemic index, protecting against oxidative stress, normalizing blood lipid levels, and contributing to reducing the incidence of cardiovascular diseases and cancer [21]. Furthermore, its richness in fiber can contribute to increasing the amount of SCFAs [22].

It is important to emphasize that these compounds are not produced in equal quantities throughout the entire intestinal tract, varying enormously depending on the tract considered. There is high production at the level of the proximal colon, decreasing proportionally as one moves from the distal colon towards the rectum. This is because the vast majority of undigested compounds in the small intestine will naturally be found immediately after the ileocecal valve and, therefore, in the proximal colon, where most of the fermentative action takes place and, consequently, where bacterial phyla capable of carrying it out will be most represented [23].

After their formation, the fate of SCFAs, and particularly the three most important ones, is not the same. Butyrate, in fact, is the main energy source for enterocytes, so it is not surprising that a large part (~70%) of the amount absorbed by the intestinal barrier remains at the level of colon cells, and only a portion of the compound passes into the periphery. The destiny of propionate is different; after colonic absorption, it passes in large quantities through the portal vein to the liver, where it carries out most of its functions, contributing to reducing lipogenesis and lowering serum cholesterol levels; meanwhile, acetate (the fatty acid produced in greater quantities) passes almost entirely to the liver and then into the peripheral circulation [24]. However, despite the fact that only a small portion of propionate and especially butyrate reaches the periphery and, thus, the organs, there are numerous functions for which they are responsible. Butyrate, for example, plays an important metabolic role by increasing fat oxidation and fasting and postprandial plasma concentrations of PYY; evidence also suggests its role in improving insulin sensitivity [25]. Consequently, there are many problems in case of their absence or low production, with many occuring during oncological treatments due to the decrease in bacterial phyla.

## 5. The Action of the Microbiota and Butyrate on Oncological Therapies

As highlighted in some of our previous publications, the impact of the microbiota on various oncological therapies is now widely recognized, underscored by a fundamental influence in terms of both therapy efficacy and the modulation of side effects [26].

In terms of conditioning effectiveness, we recall some of the most well-known examples related to chemotherapy, highlighting how the main way in which the microbiota alters the response to a chemotherapeutic agent is through the induction of chemoresistance, i.e., the ability of certain bacterial strains to modulate, transform, metabolize, or change the chemotherapy agent itself, gradually reducing its effectiveness [27]. One of the most well-known examples in this regard is related to gemcitabine and its early inactivation by certain bacterial groups (in this case, proteobacteria). In this regard, a study published in *Science* in 2017 [28] shows how resistance to chemotherapy treatment with gemcitabine in patients with pancreatic cancer is more prevalent in those with a microbiota abnormally rich in proteobacteria; it highlights how the restoration of microbial eubiosis, particularly with a decrease in proteobacteria obtained through specific antibiotic therapy, increased the therapeutic response and, thus, improved the prognosis of these subjects. Even more significant and well known in this regard is the involvement of *Fusobacterium nucleatum* in chemoresistance in colorectal tumors, through the upregulation of autophagy [29,30], and, thus, its impact on both the prognosis and the onset of the disease.

Even in terms of the toxicity of oncological treatments, the impact of the microbiota is not negligible at all. One of the clearest pieces of evidence in this regard is the ability of certain bacterial groups capable of producing a specific enzyme (β-glucuronidase) to de-conjugate certain chemotherapeutic agents (such as irinotecan), making them ready for post-treatment elimination, putting them back into circulation, and effectively increasing their concentration and, therefore, their undesirable effects [31]. However, the most comprehensive example to understand the toxic effects induced by certain microbial regimes is that related to chemotherapy-induced mucositis. Mucositis is a complication of various oncological therapies, such as radiotherapy and chemotherapy [32]. It is characterized by highly debilitating symptoms, such as nausea, abdominal cramps, bloating, and especially high-grade diarrhea [33]. The presence, or rather the absence, of certain bacterial groups (particularly those capable of producing SCFAs, especially butyrate) generates a microenvironment that directly (through the stimulation of particular receptors called TLR4) and indirectly (by increasing bacterial permeability and, therefore, the translocation of Gram-negative bacteria and LPS into circulation) favors the genesis of inflammatory and immune processes responsible for the typical toxic effects of mucositis [34]. The release of LPS induces an inflammatory response in the gastrointestinal tract, primarily mediated by the activation of the NF-kB transcription factor and the subsequent release of pro-inflammatory cytokines. This process also involves various immune cells, including macrophages and dendritic cells, which detect and respond to the presence of LPS in the gastrointestinal tract.

In addition to chemotherapy and radiotherapy, numerous other oncological treatments are now being used in the battle against these diseases. As highlighted earlier, the use of immunotherapy is becoming increasingly common and valuable. Like the treatments described previously, this scenario cannot and will not overlook our understanding of the impact that the microbiota has on immunotherapeutic treatments and our ability to modulate it.

## 6. The Action of the Microbiota and Butyrate on Immunotherapy

The impact of the gut microbiota on the response to CPIs has been primarily studied in mice, with the publication of a couple of papers that later became reference points for all scientific research in this field. In 2015, Sivian et al. [35] first demonstrated in *Science* how the microbial composition, particularly the abundance of *Bifidobacteria*, influenced the response to immune treatment with anti-PD-L1 (programmed death protein-1 ligand). In the same year, Vetizou et al. [36] highlighted in *Science* how the abundance of Bacteroidetes was crucial for the action of anti-CTLA-4 (cytotoxic T-lymphocyte antigen-4). These highly important studies paved the way for a multitude of works on this specific target, all agreeing on the role of the microbiota in patients undergoing immunotherapy [37,38,39,40]. Obviously, the bacterial composition influences the treatment response based on its characteristics and functions, and it appears quite evident that one of the fundamental and discriminating elements for this influence is the level of SCFAs produced, particularly butyrate. Indeed, among the many actions of butyrate [24] (e.g., action on the intestinal barrier, energy production for enterocytes, participation in maintaining glycemic homeostasis, inhibition of histone deacetylase (HDAC)), it also plays a crucial regulatory role in immune system function and, thus, in the immune response to carcinogenic processes. Feitelson et al. [41] suggested that SCFAs, particularly butyrate, can influence gene expression and cancer-associated signaling pathways, promoting cellular differentiation and apoptosis in tumor cells, as well as reducing the inflammation often associated with cancer development. There is now abundant scientific evidence available regarding how disturbances in the intestinal microbiota, particularly affecting the quota of butyrate-producing bacteria, impact both intestinal pathophysiology and the genesis of significant inflammatory effects, contributing to the onset of important pathologies, such as IBD [42], and the ability to maintain an active and balanced immune response.

**Figure 3:**
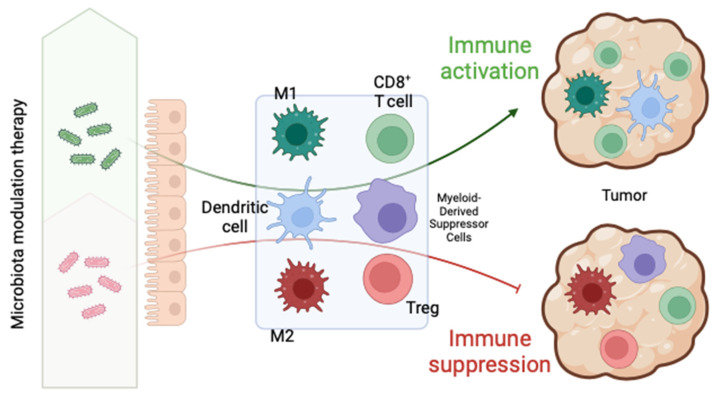
Microbiome immunomodulation through innate and adaptive immunity [39].

Butyrate plays a crucial role in enhancing the innate immune response and inflammatory response by promoting cellular activation and differentiation through its receptors (GPR109, GPR41, and GPR43), particularly macrophage differentiation, increasing their antitumor capacity [43]. These receptors are widely expressed in the myeloid cell population, including macrophages. Particularly, butyrate is known to favor the activation of M1 macrophages, with their potent immune action over M2 macrophages, which have the opposite effect [44] (Figure 3). In addition to this effect on innate immunity, butyrate has now demonstrated a fundamental impact on adaptive immunity as well; it has the capacity to both promote the differentiation of T cells into regulatory T cells (Tregs), which are important to maintaining immune homeostasis and suppressing excessive immune responses, and directly increase the response mediated by T cells (CD8+), including antitumor activity [45,46,47]. Thus, butyrate can finely regulate the immune system. On the other hand, myeloid-derived suppressor cells (MDSCs) are cells with immunosuppressive activity that act by regulating immune cells such as T lymphocytes, Tregs, and macrophages. Their elevated presence is associated with a poor response to immunotherapy.

**Figure 4:**
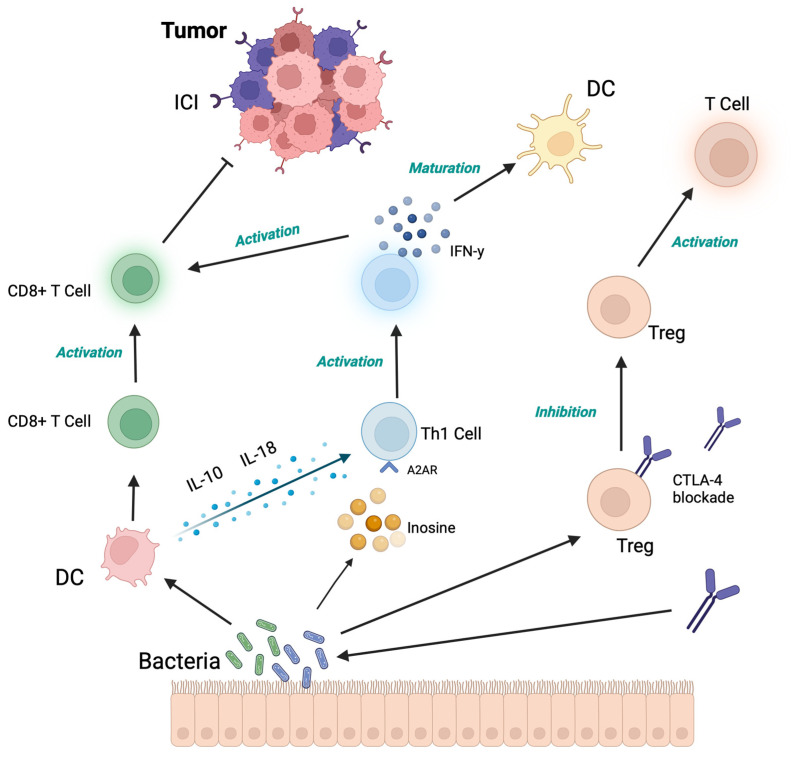
Modulation mechanism of the microbiota on CPI efficacy through CD8+ maturation, TH1 regulation with increased levels of IFN-γ, and Treg regulation, consequently activating effector T cells [48].

In summary, the action of our intestinal immune system is generally considered suppressive, capable of maintaining the delicate balance between tolerance towards commensal bacteria and intolerance towards pathogens, typically leaning towards the former condition. This dual capacity appears increasingly linked to the presence of SCFAs. In fact, the immunosuppressive mechanism is essential for maintaining intestinal homeostasis, achieved through the production of IL-18 (an interleukin with a strong anti-inflammatory and anticarcinogenic action), the activation of IL-10 (known as the interleukin of tolerance, also with a strong anti-inflammatory action), and the generation of Tregs (regulatory T cells of the immune response). All of this is facilitated by butyrate’s action on its specific receptor, called GPR109A, which promotes anti-inflammatory activity and induces the release of IL-18 in the colon epithelium. Interestingly, butyrate also participates in and regulates the opposite action, i.e., intolerance towards pathogens; its presence allows the immune system to recognize and eliminate non-self-pathogens by activating T cells (CD8+ T cells) and regulating various pathways, such as HDAC, mTORC1, and Th17 [19] (Figure 4). All of this confirms how, through these and other mechanisms, butyrate-producing bacteria are closely related to the activity of the immune system, suggesting that their presence during oncological immunotherapy treatments (aimed at activating and stimulating the immune system against tumor cells) could indeed amplify the therapeutic response and, thus, improve disease outcomes.

## 7. The Importance of Increasing Circulating Butyrate and Its Impact on Immunotherapy in Oncological Treatments

As highlighted, it is obvious how our ability to increase the quota of SCFAs (and particularly butyrate) in our body could greatly help in enhancing the efficacy of immunotherapeutic oncological treatments, which also exhibit a significant portion of non-responders and important resistance phenomena [49,50]. As explained in the formation process, there are essentially two ways to boost the amount of circulating butyric acid, both oh which are focused on enhancing its natural production: The first approach, and perhaps the most straightforward, involves stimulating increased production of this compound by providing more fermentable material to the bacteria responsible for its synthesis. This can be achieved through specific dietary choices, particularly by consuming foods rich in prebiotics [51,52]. Increasing the amount of fermentable material for butyrate production through the diet might not be effective if there is a decrease in the specific bacterial groups responsible for SCFA formation. Essentially, providing more “food” to these bacteria becomes pointless when they are depleted due to ongoing cancer treatments.

Various applications have considered the use of nutraceuticals and probiotics for the resolution of specific conditions, such as adjunct therapy in the eradication of *Helicobacter pylori* [53] or adjunct therapy to conventional therapies for the treatment of diverticular disease [54], demonstrating significant potential in the management of various digestive problems and showing other innovative applications of adjunct therapies for respiratory, gastric, and atopic diseases [55,56,57,58].

At this point, the second approach to this issue involves modulating the microbiota to enhance the presence of butyrate-producing bacterial groups, which is likely to be more effective. This centers around our capacity to cultivate and consume probiotics specifically designed for this purpose. These probiotics are bacterial strains that, when introduced into our system, can establish themselves and generate enough SCFAs and butyrate. However, the practical implementation of this idea faces significant challenges. Most bacteria suitable for this task are delicate and challenging, if not impossible, to cultivate on a large scale. Nevertheless, there are some notable exceptions, with one of the most prominent being the bacterial genus “*Clostridium butyricum*”, specifically the strain known as CBM588 (*Clostridium butyricum* MIYAIRI 588), which appears to be well suited for this task [59,60]. This is one of the most extensively studied strains due to its versatility, and it currently represents the only cultivable and usable strain due to the volume of data confirming its safety. *Clostridium butyricum* 588 is a widely recognized beneficial symbiotic bacterium, Gram-positive butyrate producer, and obligate anaerobe capable of forming spores; it is commonly found in numerous environments, with a notable presence in soil. *Clostridium butyricum* CBM588 demonstrates remarkable beneficial properties due to the production of short-chain fatty acids (SCFAs), particularly butyric acid; it has been the subject of extensive studies in the field of oncology for its ability to improve intestinal health, increase treatment tolerability, and reduce toxicity. Additionally, it can enhance the host’s immune system and promote the growth of beneficial bacteria, such as Bifidobacteria [26,61].

CBM588 is detectable in approximately 20% of adults [62] and is obviously present at the colonic level, where it ferments undigested carbohydrates, producing butyric acid. Extensive scientific research exists regarding this bacterium, as it is already extensively utilized in Eastern countries such as Japan, Korea, and China as a safe and effective treatment for various gastrointestinal issues, especially stubborn diarrhea and colitis induced by antibiotics [63]. CBM588 exhibits typical beneficial traits of a butyrate-producing bacterium, including promoting mucin production for intestinal wall protection, enhancing tight junctions (which are crucial in preventing diarrhea), and regulating inflammatory and immune responses [64].

## 8. The Clinical Action on Immunotherapy

The relationship existing between a certain type of microbiota and the efficacy of oncological immunotherapy is now a scientifically consolidated fact. There have been numerous reviews, as mentioned above, confirming how the response to immunotherapy is strongly dependent on the quota of butyrate-producing bacteria and circulating butyrate present [65]. One of the most important works in this sense was published by Frenkel et al. in 2019 [38], where the microbiota–immunotherapy relationship was certified and the main bacterial groups that correlated with an increase in immunotherapeutic action were identified, all of which were practically butyrate-producing bacteria (Table 1). The following year, in *JAMA Network Open*, Nomura et al. highlighted how among patients treated with nivolumab and pembrolizumab for various solid tumors, those who responded to treatment had a much higher concentration of SCFAs compared to non-responders, particularly their butyrate quota, which was more than double [66]. Many types of tumors were explored in these studies, highlighting how butyrate activity can transversely increase the efficacy of immunotherapy in various oncological contexts and stages. In 2018, Gopalakrishnan demonstrated how patients with melanoma undergoing immunotherapy treatment responded very differently depending on their microbial composition. Specifically, responders had a microbiota rich in some of the major butyrate producers, while non-responders carried an “unfavorable” microbiota mainly composed of Bacteroidales [67]. This work followed and confirmed the findings of the one published the previous year with ipilimumab (anti-CTLA-4) in patients with metastatic melanoma, where the response to therapy differed depending on the presence or absence of a “favorable” microbiota [68]. Melanoma is not the only tumor to have been studied in this regard and shown to be sensitive to circulating butyrate levels. In 2022, an important randomized study was published in *Nature Medicine*, comparing the efficacy of immunotherapy treatment (nivolumab and ipilimumab) with butyrate supplementation through the administration of a probiotic containing *Clostridium butyricum* (a butyrate-producing bacterial strain) vs. placebo in patients with metastatic renal carcinoma [69]. Analogous results have also been published on lung carcinoma [70].

## 9. Future Strategies

Based on what has been discussed, it is clear that oncological immunotherapy is strongly influenced by the composition of the patient’s microbiota, and that this can be exploited in predictive terms (knowing in advance which patients are likely to respond better to immunotherapy), as a sort of oncotype for immunotherapy, and in prognostic terms, since our ability to interfere with or modulate the microbiota can influence the clinical outcome. In this regard, many preclinical studies have shown that fecal microbiota transplantation (FMT) from responder animals to non-responder animals was able to improve the outcomes of the latter [67], and the first studies on patients are beginning to become available [71]. However, while FMT is still considered to be a somewhat complicated and difficult technique to execute, at least for large numbers of patients, the level of circulating butyrate can be increased through a couple of clinically feasible strategies: by using suitable prebiotics to modulate the microbiota in a eubiotic direction, favoring the implantation and colonization of symbiotic bacteria and/or SCFA producers [51,52], or by directly administering butyrate-producing bacteria. In this sense, the presence of CBM588 provides us with a tool that is easy to use, is effective, and is safe with regard to improving the response of oncological patients [59,60]. As already highlighted in one of our recent publications [61], CBM588 finds a natural place in clinical oncological practice, particularly in reducing the side effects of chemoradiotherapy (primarily mucositis and subsequent diarrhea), but its qualities in stimulating and activating the immune system also make it an ideal compound for attempting to increase the effectiveness of these treatments. Indeed, its undeniable ability to act protectively towards the intestinal barrier and thus provide a “favorable” microbiota, along with its high capacity for butyrate production, the stimulation of particular bacterial groups fundamental for immune activity (e.g., Bifidobacteria), and direct immunoregulatory and immunostimulatory action potentially, make it the ideal compound for this purpose, able to be administered both before starting oncological treatment and during the treatment in case of non-response.

## Figures and Tables

**Table 1:** Bacterial species associated with improved treatment with CPIs [38].

Cancer Type	ICT	Bacterial Species
Melanoma	Anti-CTLA-4	*Faecalibacterium prausnitzii* 12-6, *Gemmiger formicilis* ATCC27749, butyrate-producing bacteria SS2-1, *Ruminococcus*, *Lachnospiraceae*, *Clostridium XIVa*, *Blautia*
Melanoma	Anti-PD1 + anti-CTLA-4	*Faecalibacterium prausnitzii*, *Bacteroides thetaiotamicron*, *Holdemania filiformis*, *Bacteroides caccae*
Melanoma	Anti-PD1	*Faecalibacterium prausnitzii*, *Ruminococcus bromii*, *Porphyromonas pasteri*, *Clostridium hungati*, *Phascolarctobacterium faecium*
Melanoma	Anti-PD1	*Enterococcus faecium*, *Collinsella aerofacients*, *Bifidobacterium adolescentis*, *Klebsiella pneumoniae*, *Veillonella parvula*, *Parabacteroides merde*, *Lactobacillus* sp., *Bifidobacterium longum*
NSCLC, RCC	Anti-PD1	*Akkermansia muciniphila*, *Lachnospiraceae*, *Erisypelotrichaceae lacteria* 5-2-64, *Enterococus faevium*, *Alistipes indistinctus*, *Bacteroidaceae*, *Bacteroides xylanisolvens*, *Bacteroides nordii*

Abbreviations: ICT, immune checkpoint inhibitor therapy; CTLA, cytotoxic T-lymphocyte-associated protein 4; PD1, programmed death receptor-1; NSCLC, non-small-cell lung carcinoma; RCC, renal cell carcinoma.
